# Omega-3 Fatty Acids in Erythrocyte Membranes as Predictors of Lower Cardiovascular Risk in Adults without Previous Cardiovascular Events

**DOI:** 10.3390/nu13061919

**Published:** 2021-06-03

**Authors:** Gustavo Henrique Ferreira Gonçalinho, Geni Rodrigues Sampaio, Rosana Aparecida Manólio Soares-Freitas, Nágila Raquel Teixeira Damasceno

**Affiliations:** Department of Nutrition, School of Public Health, University of São Paulo, São Paulo 01246-904, Brazil; ghfg93@gmail.com (G.H.F.G.); genirs@usp.br (G.R.S.); rosanaso@usp.br (R.A.M.S.-F.)

**Keywords:** *n*-3 polyunsaturated fatty acids, cardiovascular risk estimates, cardiovascular diseases, biomarkers, cardiovascular risk factors

## Abstract

*Background:* This study investigated the association of omega-3 polyunsaturated fatty acids (*n*-3 PUFA) within erythrocyte membranes and cardiovascular risk assessed by three different estimates. *Methods:* Inclusion criteria were individuals of both sexes, 30 to 74 years, with at least one cardiovascular risk factor, and no previous cardiovascular events (*n* = 356). Exclusion criteria were individuals with acute or chronic severe diseases, infectious diseases, pregnant, and/or lactating women. Plasma biomarkers (lipids, glucose, and *C*-reactive protein) were analyzed, and nineteen erythrocyte membrane fatty acids (FA) were identified. The cardiovascular risk was estimated by Framingham (FRS), Reynolds (RRS), and ACC/AHA-2013 Risk Scores. Three patterns of FA were identified (Factor 1, poor in *n*-3 PUFA), (Factor 2, poor in PUFA), and (Factor 3, rich in *n*-3 PUFA). *Results:* Total cholesterol was inversely correlated with erythrocyte membranes C18:3 *n*-3 (r = −0.155; *p* = 0.004), C22:6 *n*-3 (r = −0.112; *p* = 0.041), and total *n*-3 (r = −0.211; *p* < 0.001). Total *n*-3 PUFA was associated with lower cardiovascular risk by FRS (OR = 0.811; 95% CI= 0.675–0.976). Regarding RRS, Factor 3 was associated with 25.3% lower odds to have moderate and high cardiovascular risk (OR = 0.747; 95% CI = 0.589–0.948). The ACC/AHA-2013 risk score was not associated with isolated and pooled FA. *Conclusions:* *n*-3 PUFA in erythrocyte membranes are independent predictors of low-risk classification estimated by FRS and RRS, which could be explained by cholesterol-lowering effects of *n*-3 PUFA.

## 1. Introduction

Cardiovascular diseases (CVD) remain the major cause of death worldwide. Therefore, the assessment and monitoring of cardiovascular (CV) risk through algorithms has shown to be an accurate tool to predict outcomes, as well as to improve treatment indication when compared with the isolated use of risk factors [1,2,3]. The estimates use risk factors that are the major contributors to cardiovascular events (i.e., age, sex, glycemia, blood pressure, and blood lipids) [3,4,5]. The ten-years CV risk estimation is relevant especially in moderate-risk patients because the intuitive ten-year period is important in making practical and usually therapeutic, decisions. Cardiovascular risk assessment models have been built to guide the treatment of modified cardiovascular risk factors and, in the last decade to help therapeutic goals based on statins. Moreover, the estimates provide insight into the individual contribution of variables to the patient’s risk, guiding the preventive care [1]. However, the application of these estimates requires previous validation for the target population. Many CV risk estimates were developed based on American or European white populations, and the estimation of multi-ethnic populations is often overestimated [6,7,8,9]. Nevertheless, the Framingham Risk Score (FRS) is the most popular estimating tool and its use is currently recommended by many guidelines, including in Brazil [10]. 

Omega-3 polyunsaturated fatty acids (*n*-3 PUFA) are often highlighted due to several mechanisms that modify CV risk factors, slow down the atherosclerotic process and, possibly change cardiovascular events. The eicosapentaenoic (EPA; C20:5 *n*-3) and docosahexaenoic acids (DHA; C22:6 *n*-3) are the main components of this family, is often linked to antiarrhythmic effects, autonomic function improvement, decreased platelet aggregation, vasodilatory effects, blood pressure reduction, endothelial function improvement, atherosclerotic plaque stabilization, increased adiponectin synthesis, reduction of collagen deposition in the arteries, anti-inflammatory effects, and reduction of plasma triglycerides and cholesterol, consequently reducing CVD risk [11]. Despite that, reports of randomized trials have shown small or even null effects on cardiovascular risk factors and outcomes [12]. 

Most of the studies show methodological differences and do not assess *n*-3 PUFA biomarkers. Circulating or tissue *n*-3 PUFA have proven their superiority in estimating habitual intake compared to dietary assessment [13]. Based on that, previous studies have associated *n*-3 PUFA in erythrocyte membranes with reduced CV risk and mortality [13,14,15,16]. Because *n*-3 PUFA alter some components included in CV risk estimates, it is possible to state that *n*-3 PUFA influence the overall CV risk which is frequently used to guide preventive care. Thus, the nutritional status of *n*-3 PUFA may be useful in CVD prevention. However, as far as it is known, no previous study investigated the association of isolated and clustered FA biomarkers with different cardiovascular risk estimates.

Therefore, the main goal of this study was to investigate the association of erythrocyte membranes *n*-3 PUFA with different cardiovascular risk estimate classifications in Brazilian individuals. In addition, we also evaluated the association of modified CV risk factors used in estimates with isolated and clusters *n*-3 PUFA.

## 2. Materials and Methods

### 2.1. Study Design and Participants

This was a cross-sectional study, using the baseline data from the CARDIONUTRI clinical trial (ReBEC: RBR-2vfhfv), which included individuals from the outpatient clinic at the University Hospital of the University of São Paulo. The study selection was made public by poster, newspaper, and digital media (sites, electronic mailing, and social networks). Inclusion criteria were individuals of both sexes, 30 to 74 years, with at least one cardiovascular risk factor, and no previous cardiovascular events. Exclusion criteria were individuals with acute or chronic severe diseases, infectious diseases, pregnant, and/or lactating women. Individuals interested in participating in the study were submitted to a short phone interview to assess inclusion and exclusion criteria. Additionally, individuals were submitted to electrocardiogram assessment by a trained physician, and those with alterations suggesting previous cardiovascular events were excluded. Three hundred and seventy-four individuals were recruited for the study from 2011–2012. Two individuals declined after clarification of the study design. Fourteen were excluded due to altered electrocardiogram and one due to recent HIV diagnosis. At the end of the recruitment, 356 individuals were included in the study.

### 2.2. Clinical, Physical Activity, and Diet Assessment

Sociodemographic status, lifestyle, family history of chronic diseases, self-report of non-communicable chronic diseases, and current medication use were investigated through questionnaires. Physical examination included body mass index (BMI) assessment and blood pressure levels. Dietary intake was obtained through three 24 h-recalls and assessed in the Food Processor software (ESHA Research, 2012), with subsequent energy adjustment [17]. A physical activity questionnaire validated for the Brazilian population was applied [18,19,20]. This questionnaire investigates the habitual physical activity (divided into physical exercise in leisure, leisure, and locomotion activities and total physical activity score) performed in the last 12 months, associated with frequency, duration, intensity. Baecke’s physical activity scores do not allow to classify physical activity, however, for each one of its sixteen questions, the points vary from 0 (zero) to 5. The final score is directly proportional to physical activity and is useful to associate with health outcomes [18,19,20].

### 2.3. Biochemical Measurements

Blood was drawn after a 12-h fast, placed in EDTA tubes (1.0 mg/mL), and erythrocytes were separated from plasma by centrifugation, and both were frozen at −80 °C immediately after collection. Protease inhibitors (10 µg/mL of aprotinin, 10 µg/mL of benzamidine and 5 µg/mL of phenylmethylsulfonyl fluoride) and BHT (100 µg/mL) were added to preserve samples. All samples were divided into aliquots to avoid repeated defrost cycles and storage at −80 °C until analyses. Plasma total cholesterol, HDL-c, TG, glucose (Labtest Diagnostica SA, MG, Brazil), Apo A-I and Apo B (Wako Chemicals USA Inc., Richmond, VA, USA), and high sensitivity *C*-reactive protein (hs-CRP) (Diagnostic System Laboratories, Inc., Webster, TX, USA) were measured by commercial kits. LDL-c was calculated according to the Friedewald equation. 

### 2.4. Erythrocyte Fatty Acids Analysis

The analysis of FA from erythrocyte membranes was performed based on a previous method [21]. After plasma separation (3000× *g*, 10 min, 4 °C), 300 μL of erythrocytes were washed with 5 mL of phosphate-buffered saline (PBS) solution (pH 7.4) four times. The precipitate was transferred to threaded tubes, to which 1.75 mL of methanol, 50 μL of an internal standard solution containing 1 mg tridecanoic acid (C13:0)/1 mL hexane, and 100 μL of acetyl chloride were added. Thereafter, the solution was vortexed and heated in a water bath at 90 °C for 1 h. After that, 1.5 mL of hexane was added, and the solution was homogenized for 1 min. The samples were centrifuged at 1500× *g*, 4 °C for 2 min, and 800 μL of the supernatant was transferred to a different tube. This step was repeated with the addition of 750 μL of hexane. The tubes containing the collected supernatants were placed on a centrifugal concentrator at 40 °C for 20 min. Then the FA methyl esters were dissolved in 150 μL of hexane and transferred to a glass insert in a vial. Analyses were conducted considering the fatty acids individually, as well as the total *n*-3 (C18:3 *n*-3 + C20:3 *n*-3 + 20:5 *n*-3 + C22:5 *n*-3 + 22:6 *n*-3), total *n*-6 (C18:2 *n*-6 + C20:4 *n*-6) and Omega-3 Index (C20:5 *n*-3 + C22:6 *n*-3), the latter having been named by Harris and von Schacky [13]. To assess biological effects of fatty acids, the following ratios were calculated: C20:4 *n*-6/C20:5 *n*-3, C18:3 *n*-3/C20:5 *n*-3, C18:3 *n*-3/C22:6 *n*-3 and C18:2 *n*-6/C18:3 *n*-3.

### 2.5. Cardiovascular Risk Assessment

The CV risk was assessed by FRS [1,22], Reynolds Risk Score (RRS) [23,24], and the American College of Cardiology/American Heart Association 2013 Risk Score (ACC/AHA-2013) [25]. The CV risk was stratified into three categories for each score: low, moderate, and high risk. Diabetes (i.e., glucose ≥ 126 mg/dL or current hypoglycemic medication use) was considered a coronary artery disease (CAD) equivalent [26]. 

### 2.6. Statistical Analysis

Distribution of variables was assessed through the Kolmogorov-Smirnov test. Sample characteristics are presented as mean and standard deviation (SD) or median and interquartile range (IQR) depending on the variable’s distribution. For categorical variables, results are shown in absolute value (*n*) and its percentage (%). Spearman’s and Pearson’s correlations were applied to evaluate associations between cardiovascular risk factors and FA.

Kappa (k) agreement analysis was performed between ACC/AHA 2013, FRS, and RRS to verify the agreement between the cardiovascular risk stratifications, and the strength of agreement was classified according to Landis and Koch (1977) [27]. 

A factor analysis was performed to establish the patterns of erythrocyte membranes FA composition to subsequently associate them with CV risk. It is a multivariate statistical analysis for the identification of factors in a set of measurements [28]. Sample adequacy was checked using the Kaiser-Meyer-Olklin (KMO) index and Barlett’s test of sphericity. KMO values > 0.50 and *p* < 0.05 were considered acceptable. The choice of the number of factors was based on eigenvalues > 1.0 and scree plot analysis. Factor loadings were analyzed after orthogonal rotation using the varimax method. The considered threshold of factor loadings was 0.2. Negative loadings indicated that FA were inversely associated with the corresponding factor, just as positive loadings indicated a direct association [28]. Three factors were generated. 

To further evaluate potential confounders of the associations between erythrocyte membranes FA and CV risk estimates, multiple linear and logistic regressions were applied using baseline sample characteristics as covariates (age, sex, race, schooling, smoking, systolic blood pressure, BMI, glucose, triglycerides, total cholesterol, HDL-c, *C*-reactive protein, physical activity, drinking habits, treatments with statins, antihypertensives, fibrates, and hypoglycemic drugs, family history of myocardial infarction, obesity, hypertension, and stroke) and total *n*-3 and *n*-6 PUFA, Factor 1, Factor 2 and Factor 3 as dependent variables. Assumptions for linear regression such as lack of multicollinearity of predictors, residuals’ homoscedasticity and normality, linearity, and independence were evaluated. *n*-6 PUFA, Factor 1, and Factor 3 covered all assumptions, while total *n*-3 and Factor 2 presented nonparametric residuals. Thus, linear regressions were not applied to these latter variables. The multiple linear regressions were applied using the backwards method, and final models were presented. Multiple logistic regressions were applied to total *n*-3 PUFA and Factor 2 (categorized by median) using the backwards-likelihood ratio method and models with the best correct classification were chosen. 

Logistic regressions were used with CV risk scores as dependent variables (0 = low CV risk and 1 = moderate and high CV risk) and FA or Factors as independent variables. Because age, race, sex, total cholesterol, HDL-c, SBP, glucose, and *C*-reactive protein are covariates already entered into the equations of the CV risk estimates, these were not used as adjustments of the regressions. All regressions were adjusted by physical activity, BMI, and education level. Since there is no data on socioeconomic status, a known predictor of CV risk, education level was used as an adjustment in the models [29].

The missing data was handled by pairwise methods [30]. All tests were two-sided, considered significant when *p* < 0.05, and performed using the software Stata version 14 and SPSS version 20.

## 3. Results

The characteristics of the individuals (*n* = 356) are summarized in Table 1. The mean age was 52.5 (10.4) years old (men = 49.4 years and women = 54.4 years; *p* < 0.001) and 62.6% were women. It was observed a high frequency of hypertension (57%) and a family history of the disease (65.2%). In addition, 51.7% of the individuals were on antihypertensive treatment. Most individuals were classified as a high cardiovascular risk by FRS (52.2%) and ACC/AHA 2013 score (50.4%), while only 29.1% classified by RRS show similar risk levels. The mean BMI was 30.9 (5.8) Kg/m^2^. Current smoking (26.3% vs. 15.7%; *p* = 0.003) and alcohol intake (64.7% vs. 35%; *p* < 0.001) were more frequent in men. As expected, for all cardiovascular risk estimates men and women showed significant differences (Table S1). Table 2 describes the biochemical and clinical profile of individuals. The mean total cholesterol level was 205.0 (42.6) mg/dL. The mean CRP was 2.8 (1.2–6.0 mg/L). Dyslipidemia (53.9%) and hypertension (57.0%) were highly prevalent. When individuals were compared by sex, women showed higher total cholesterol, LDL-c, and CRP than men, while HDL-c and Apo A-I were higher (Table S2).

Although women had a higher intake of total lipids, eicosatrienoic (C20:3 *n*-3) and docosapentaenoic (C22:5 *n*-3) than men, the 19 FA in erythrocyte membranes presented in Table 3 did not show differences between sexes (Table S3). Fourteen from nineteen FA identified met the criteria for factorial analysis model (KMO = 0.632; Barlett’s Test of Sphericity < 0.001). Factor 1 was rich in *n*-6 PUFA and poor in *n*-3 PUFA, Factor 2 was poor in PUFA, and Factor 3 was rich in *n*-3 PUFA and poor in *n*-6 PUFA (Table S4).

Total cholesterol was inversely correlated with erythrocyte membranes C18:3 *n*-3 (r = −0.155; *p* = 0.004), C22:6 *n*-3 (r = −0.112; *p* = 0.041), Omega-3 Index (r = −0.124; *p* = 0.023) and total *n*-3 (r = −0.211; *p* < 0.001), and positively correlated with total *n*-6 (r = 0.178; *p* = 0.001) and Factor 1 (r = 0.170; *p* = 0.002), which is rich in *n*-6 PUFA and poor in *n*-3 PUFA (Table S5). Multivariate linear and logistic regressions were applied to evaluate the associations between the covariates entered in CV risk estimates, baseline characteristics and erythrocyte membranes *n*-3 and *n*-6 PUFA to verify potential confounding factors (Tables S6 and S7). Total cholesterol, BMI, triglycerides, family history of obesity, and age were independently associated with erythrocyte membranes FA (Tables S6 and S7). 

**Figure 1:**
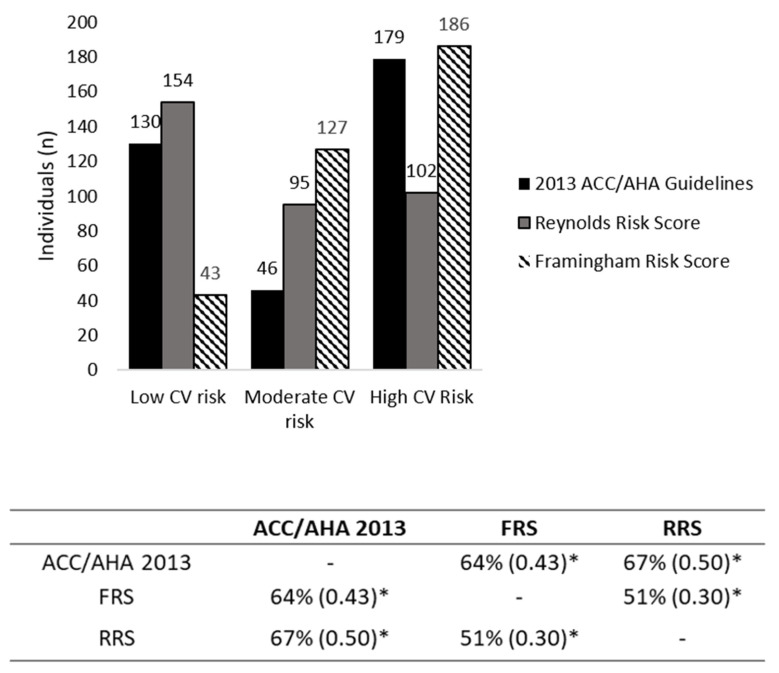
Distribution and agreement of global cardiovascular risk stratifications between different predictive equations. Data are shown in % agreement and kappa values. *: *p*-value < 0.001.

Figure 1 shows the CV risk classification and its concordance. The most frequent stratification of CV risk assessment by RRS was low CV risk (*n* = 154; 43.9%), whilst ACC/AHA 2013 and FRS were the scores that classified most individuals as high risk (*n* = 179; 50.4% and *n* = 186; 52.2%, respectively). The agreement of cardiovascular risk stratifications obtained through estimates was modest. The agreement between FRS and RRS was 51% (k = 0.30, *p* < 0.001), and there was a moderate agreement between FRS and ACC/AHA 2013, of 64% (k = 0.43, *p* < 0.001) and between ACC/AHA 2013 and RRS, with 67% (k = 0.50, *p* < 0.001). Based on that, all CV risk estimates were maintained in the next analyses.

The Table 4 describes the association of CV scores estimates and erythrocytes membranes FA. The regression analyses showed that each unit increase of C18:3 *n*-3 was associated with 20.8% odds reduction of being classified as intermediate or high risk (OR = 0.792; 95% CI = 0.635–0.988). Each unit increase of total *n*-3 PUFA (C18:3 *n*-3 + C20:5 *n*-3 + C22:6 *n*-3) had 20.2% odds increase of low CV risk classification by FRS (OR = 0.798; 95% CI = 0.672–0.946). There were also 2.8% odds increase of low CV risk classification regarding the C18:3 *n*-3/C20:5 *n*-3 ratio (OR = 0.972; 95% CI = 0.945–1.000). Each unit increase of *n*-6/*n*-3 and C18:2 *n*-6/C18:3 *n*-3 ratios were associated with 47.3% (OR = 1.473; 95% CI = 1.021–2.126) and 27.6% (OR = 1.276; 95% CI = 1.043–1.561) odds increase of intermediate or high CV risk classification, respectively. After adjustment, only total *n*-3 PUFA remained statistically significant (OR = 0.811; 95% CI = 0.675–0.976). 

Factor 1 (rich in *n*-6 PUFA) was associated with odds increase of intermediate or high CV risk classification by 40.8% by FRS (OR = 1.408; 95% CI = 1.036–1.913). After adjustment, the odds increased to 46.9% (OR = 1.469; 95% CI = 1.056–2.043) by FRS and 27.6% by RRS (OR = 1.276; 95% CI: 1.010–1.612). Factor 3 (rich in *n*-3 PUFA) was associated with odds increase of low CV risk classification by 26.6% according to RRS (OR = 0.734; 95% CI = 0.585–0.921). After adjustment, the odds increased by 25.3% (OR = 0.747; 95% CI = 0.589–0.948). Erythrocyte membranes FA and membranes patterns were not statistically significant associated with the ACC/AHA 2013 risk score (Table S8).

## 4. Discussion

The findings of the study show that a higher content of *n*-3 PUFA in erythrocyte membranes was associated with higher odds of CV risk being classified as low. Unlike prior studies, the patterns of erythrocyte membranes FA composition were investigated, and the results corroborate the cardioprotective associations of *n*-3 PUFA. 

CV risk estimates do not allow to establish a causal relationship with cardiovascular events and mortality but are useful for healthcare professionals to monitor interventions focused on modifying classic risk factors and to further assess individual patient risk. Regarding the effects of *n*-3 PUFA on CV risk factors by multiple direct and indirect mechanisms, it is plausible to assume it influences the estimated CV risk as proposed by previous studies. In 50 cases with acute non-fatal MI and 50 age- and sex-matched controls without MI the Omega-3 Index was significantly lower in cases than in controls (9.57% (SEM = 0.28) vs. 11.81% (SEM = 0.35); *p* < 0.001) in addition to the decreased risk of non-fatal MI (OR = 0.08; 95% CI = 0.02–0.38). Also, a CV risk estimate based on the FA profile (sum of C20:5 *n*-3, C18:3 *n*-3, *trans*-oleic acid, and C20:4 *n*-6) showed a higher contribution to the discrimination of MI cases compared to controls when compared to FRS, being a potential predictor of outcomes [31]. The similar way, a study evaluating MI 2-year mortality showed that the red blood cells (RBC) FA C20:5 *n*-3 and C22:5 *n*-6 of 1144 patients changed the *c*-statistic of the GRACE score from 0.747 (*p* < 0.001) to 0.768 (*p* < 0.05 vs. GRACE alone), improved the net reclassification index by 31% (95% CI = 15–48%) and the relative incremental discrimination index by 19.8% (95% CI = 7.5–35.7%). Those results show that RBC FA improved the prediction of 2-year mortality over the GRACE score in MI patients [32]. In the present study, two patterns of erythrocyte membranes FA composition were associated with CV risk classification. The pattern rich in *n*-3 PUFA (Factor 3) increased the odds of low-risk classification by 25.3% by RRS, whilst the pattern rich in *n*-6 PUFA (Factor 1) increased the odds of moderate or high-risk classification by 46.9% and 27.6% by FRS and RRS, respectively. 

Studies have shown inverse associations between *n*-3 PUFA biomarkers and CVD. Two meta-analyses have shown associations of *n*-3 PUFA biomarkers from different compartments with coronary risk reduction [14,15]. In a cohort, C20:5 *n*-3, C22:6 *n*-3, and Omega-3 Index in erythrocyte membranes were inversely associated with CV mortality, with stronger results when C20:5 *n*-3 was higher than 1% [33]. In several populations, the Omega-3 Index is associated with reduced coronary risk [13,34,35]. Recently, in the Framingham Offspring Cohort, individuals with Omega-3 Index higher than 6.8% had 39% fewer cardiovascular events compared to those in which the index was lower than 4.2%. Another finding of this study was 59% and 32% lower risks of stroke and all-cause mortality in individuals with C22:6 *n*-3 higher than 5.96% when compared to those lower than 3.69% [16]. The present study did not identify a significant association of the Omega-3 Index and CV risk classifications; however, robust associations were observed for C18:3 *n*-3 and total *n*-3 with lower CV risk estimated by FRS and pooled FA rich in *n*-3 (Factor 3) and RRS. Conversely, Factor 1 (rich in *n*-6), and total *n*-6/*n*-3 and C18:2 *n*-6/C18:3 *n*-3 ratios modified the previous association, reducing the benefits attributed to FA *n*-3. This profile can be explained by the reduced content of the Omega-3 Index (<4%) in 62.7% of the participants, whereas 36.4% had a sub-optimal content (4% to 8%), with only 0.9% showing an optimal level, according to Omega-3 Index classification proposed by Harris & von Schacky [13]. 

The complex relationship of FA and CV risk estimates may be partially explained by associations between C18:3 *n*-3, C22:6 *n*-3, and total *n*-3 PUFA and total cholesterol, suggesting that the associations with CV risk classification are related to the cholesterol-lowering effect of *n*-3 PUFA. Zibaeenezhad et al. evaluated the impact of fresh fish intake (250 g/week) and fish oil supplementation (2 g/day) during 8 weeks on lipid profile. The consumption of dietary fish has shown better effect on the reduction of total cholesterol and LDL-c compared with fish oil [36]. Although the positive effect of *n*-3 on hypertriglyceridemia (from 25% to 30% triglycerides reduction) is a consensus in literature [37], the isolated effect on total cholesterol and LDL-c remains controversial. Two systematic reviews based on fish intake, EPA-containing capsules, and algae DHA oil confirm the positive effect of *n*-3 PUFA on the reduction of triglycerides without changes in LDL-c [38,39]. Contrary, Wei et al. (2011) observed a 5% LDL-c increase after DHA intake, while EPA decreased by 1% [40]. Together, these results indicate that *n*-3 PUFA modulates lipid profile by multiple mechanisms, which contributes to association with better CV risk classification observed in the present study.

The three CV risk estimates tested in this study were validated in American Caucasians, and the application on different populations without validation may overestimate the risk. Although the Brazilian population is multi-ethnic, the Brazilian Society of Cardiology guidelines recommends using FRS for CV risk estimation [10]. The ACC/AHA 2013 risk score is currently recommended by America Heart Association guidelines [25], and the RRS had a better prediction compared to FRS in the American population, in addition to considering family history and inflammation in the equation [23,24]. The ACC/AHA 2013 risk score has shown good calibration and discrimination in an American cohort [9], but its application in and European population indicated 96.4% of men and 65.8% of women classified as high risk [6]. In the multi-centric cohort Multi-Ethnic Study of Atherosclerosis (MESA), containing 6814 individuals self-referred as Caucasians, Blacks, Hispanics, or Chinese, the ACC/AHA 2013 risk score had the worst calibration and discrimination compared to FRS, RRS, and ATP-III Risk Score, overestimating risk for both men (154%) and women (67%), with an overall disagreement of 115% [7]. In turn, FRS overestimated men’s risk in 37% and women’s risk in 8%, with an overall disagreement of 25%, whilst RRS overestimated men’s risk in only 9%, and underestimated women’s risk in 21%, in addition to showing the slightest overall disagreement (−3%) [7]. In Women’s Health Initiative Observational Cohort, FRS overestimated the risk and had worse calibration and discrimination compared to RRS [8]. In the MESA cohort, RRS also outperformed FRS in predicting subclinical atherosclerosis assessed by coronary artery calcification (CAC), an important predictor of CV risk, through computerized tomography [41]. Those studies suggest that ACC/AHA 2013 risk score and FRS overestimate risk in multi-ethnic populations, and the frequency of high-risk stratifications in this study corroborates with them. The agreement analyses performed in this study confirm the differences between these CV risk estimates. Although FRS and ACC/AHA 2013 consider the same parameters to estimate the CV risk, the subtle differences in both algorithms may explain the modest agreement between them and subsequently, the absence of association of *n*-3 PUFA and ACC/AHA 2013 observed in this study. 

Furthermore, it important to highlight that despite the highest agreement between ACC/AHA 2013 and RRS (67%; k = 0.50), due to the high frequency of low-risk classifications, the first estimate does not consider the inflammation in CV risk. We hypothesized that because the CRP is a component of RRS and is modulated by *n*-3 PUFA, it would strengthen the association between both variables. However, no associations between CRP and *n*-3 PUFA were found in the study. Previous studies show a strong relationship between *n*-3 PUFA and CRP. In 2019, the study of Omar et al. showed that high intake of *n*-3 (2.0 g/day) reduced blood lipids (total cholesterol, LDL-c, and triglycerides) and inflammatory markers such as interleukin-6 and CRP [42]. Similar results were observed when purified eicosapentaenoic acid ethyl ester (4.0 g/day) was used in ANCHOR study, in which a significant reduction in triglycerides and CRP, without changing LDL-c [43].

Certainly, the most relevant limitation of our results is the lack of information about CV outcomes. Another limitation was the lack of socioeconomic status data in the study, which is an important predictor of CV risk. Despite of that, education level, which was used as the adjustment, may reflect socioeconomic status as predictor of health [29]. Furthermore, the modifiable and non-modifiable risk factors considered in the CV risk estimates are not able to explain all cardiovascular events, so the CV outcome prediction may not reflect the real risk. Therefore, the effects of *n*-3 PUFA on CV risk may be underestimated due to mechanisms that act independently of traditional risk factors, such as platelet inhibition and arrhythmia reduction [11,44]. It is important to note that an important portion of the individuals in the study use medications that can affect the associations and mask the effects of PUFA on CV health, such as lipid-lowering drugs. For future studies regarding CV risk scores, a prescreening of individuals based on *n*-3 PUFA level could show more clearly the effects of *n*-3 PUFA on CV risk classification, although higher levels (>4%) of *n*-3 PUFA may not be frequent in Western countries due to low intake [35].

The strengths of this investigation include the application of FA biomarkers, being more objective than the traditional dietetic assessment. This study investigated not only single FA associations but the FA patterns through factor analysis. These patterns might depict the manifold biological interactions with FA, which the isolated analysis would not do. As far as it is known, this study is the first to assess erythrocyte membranes FA patterns through factor analysis. Moreover, the application of multiple CV risk estimates uses major CV risk factors and indirectly reflects the clinical outcomes, being useful in short-term or cross-sectional investigations. 

## 5. Conclusions

In conclusion, the results of this study have shown that *n*-3 PUFA in erythrocyte membranes are associated with better CV risk classification estimated by FRS and RRS in Brazilian individuals, which could be explained by the cholesterol-lowering effects of *n*-3 PUFA. 

## Figures and Tables

**Table 1:** Demographic and clinical characterization of individuals.

Variables	*n*	Total
Age (years)	356	52.5 (10.4)
*Ethnicity (n, %)*	356	
White		238 (66.9)
Non-white		118 (33.1)
*Smoking (n, %)*	356	
Current smoker		70 (19.7)
Non-smoker		286 (80.3)
*Alcohol consumption (n, %)*	356	
Yes		164 (46.1)
No		192 (53.9)
*Education (n, %)*		
High school or less		208 (58.4)
College		148 (41.6)
*Chronic non-communicable diseases (n, %)*	356	
Diabetes Mellitus		72 (20.2)
Hypertension		203 (57.0)
Hypothyroidism		43 (12.1)
Dyslipidemia		192 (53.9)
*Medication (n, %)*	356	
Statins		98 (27.5)
Antihypertensives		184 (51.7)
Hypoglycemic		74 (20.8)
Fibrates		9 (2.5)
*Family history of diseases (n, %)*	356	
Obesity		64 (18.0)
Hypertension		232 (65.2)
Myocardial infarction		100 (28.1)
Stroke		68 (19.1)
Diabetes Mellitus		134 (37.6)
Physical activity (points)		7.18 (1.39)
*Framingham Risk Score (n,%)*	356	
Low risk		43 (12.1)
Moderate risk		127 (35.7)
High risk		186 (52.2)
*Reynolds Risk Score (n,%)*	351	
Low risk		154 (43.9)
Moderate risk		95 (27.1)
High risk		102 (29.1)
*ACC/AHA-2013 Risk Score (n,%)*	355	
Low risk		130 (36.6)
Moderate risk		46 (13.0)
High risk		179 (50.4)

Continuous variables are shown as mean (standard deviation) or median (interquartile range), and categorical data as *n* (%). BMI: body mass index; SBP: systolic blood pressure; DBP: diastolic blood pressure; TG: triglycerides; LDL-c: low density lipoprotein-cholesterol, HDL-c: high density lipoprotein-cholesterol.

**Table 2:** Biochemical and clinical characterization of individuals.

Variables	*n*	Total
SBP (mmHg)	356	133 (18.0)
DBP (mmHg) Hypertension (≥140 mmHg) (*n*, %)	356	81 (10.0) 111 (31.2)
BMI (kg/m^2^) Obesity (BMI ≥ 30.0 kg/m^2^) (*n*, %)	356	30.9 (5.8) 182 (51.1)
Total cholesterol (mg/dL) Hypercholesterolemia (≥200mg/dL) (*n*, %)	354	205.0 (42.6) 193 (54.2)
LDL-c (mg/dL) High LDL-c (≥130 mg/dL) (*n*, %)	340	137.3 (38.7) 196 (55.1)
HDL-c (mg/dL) Low-HDL-c (<40 mg/dL) (*n*, %)	354	36.0 (30.0–42.3) 125 (35.1)
Triglycerides (mg/dL) Hypertriglyceridemia (≥150 mg/dL) (*n*, %)	354	130.5 (98.0–191.3) 145 (40.7)
Glucose (mg/dL) Hyperglycemia (≥100 mg/dL) (*n*, %)	354	98.0 (91.0–108.0) 164 (46.1)
Apo A-I (mg/dL) Low-Apo A-I (<120 mg/dL) (*n*, %)	355	132.2 (25.7) 230 (64.6)
Apo B (mg/dL) High-Apo B (≥120 mg/dL) (*n*, %)	355	104.7 (24.8) 88 (24.7)
*C*-reactive protein (mg/L) High-CRP (>1.0 mg/L) (*n*, %)	347	2.84 (1.2–6.0) 275 (77.2)

Categorical variables are shown as absolute value (*n*) and frequency (%). Continuous variables are shown as mean (standard deviation) or median (interquartile range). BMI: body mass index; SBP: systolic blood pressure; DBP: diastolic blood pressure; TG: triglycerides; LDL-c: low density lipoprotein-cholesterol, HDL-c: high density lipoprotein-cholesterol.

**Table 3:** Erythrocyte membranes fatty acids profile (*n* = 335).

Variables	Total
*SFA (%)*	
C16:0	43.6 (41.1–47.5)
C18:0	24.8 (22.9–27.3)
C20:0	0.7 (0.6–0.8)
C22:0	1.1 (0.9–1.4)
C24:0	0.3 (0.1–0.7)
*MUFA (%)*	
C16:1 *n*-7	0.3 (0.2–0.6)
C18:1 *n*-9	10.0 (3.5)
C20:1 *n*-9	0.0 (0.1–0.1)
C22:1 *n*-9	0.1 (0.1–0.2)
C24:1 *n*-9	1.3 (0.5)
*PUFA n*-6 *(%)*	
C18:2 *n*-6	4.7 (1.8)
C18:3 *n*-6	0.2 (0.1–0.2)
C20:2 *n*-6	0.1 (0.1–0.2)
C20:3 *n*-6	0.6 (0.3)
C20:4 *n*-6	2.5 (1.4–5.1)
C22:2 *n*-6	0.4 (0.3–0.6)
Total *n*-6	9.4 (3.8)
*PUFA n*-3 *(%)*	
C18:3 *n*-3	0.2 (0.1–0.2)
C20:5 *n*-3	0.2 (0.1–0.3)
C22:6 *n*-3	3.4 (2.7–4.2)
Omega-3 Index	3.6 (3.0–4.5)
Total *n*-3	5.7 (4.8–6.7)
*Fatty acids ratios*	
C16:0/C16:1 *n*-7	130.7 (67.9–232.6)
C18:0/C18:1 *n*-9	2.5 (2.0–3.4)
*n*-6/*n*-3	1.7 (1.0–2.4)
C20:4 *n*-6/C20:5 *n*-3	12.9 (5.6–27.6)
C18:3 *n*-3/C20:5 *n*-3	9,1 (5.7–14.0)
C18:2 *n*-6/C20:4 *n*-6	1.8 (1.0–2.8)
C18:2 *n*-6/C18:3 *n*-3	2.4 (1.4–4.2)

SFA: saturated fatty acids; PUFA: polyunsaturated fatty acids; MUFA: monounsaturated fatty acids. Omega-3 Index: C20:5 *n*-3 + C22:6 *n*-3. Total *n*-3: C18:3 *n*-3 + 20:5 *n*-3+ 22:6. *n*-6: C18:2 *n*-6 + C18:3 *n*-6 + C20:2 *n*-6 + C20:3 *n*-6 + C20:4 *n*-6 + C22:2 *n*-6. Data are shown as mean (standard deviation) or median (interquartile range) depending on the distribution.

**Table 4:** Logistic regression models of isolated and pooled erythrocyte membranes fatty acids and FRS and RRS.

Fatty Acids	Framingham Risk Score				Reynolds Risk Score			
	Unadjusted Model		Adjusted Model *		Unadjusted Model		Adjusted Model *	
	OR	CI (95%)	OR	CI (95%)	OR	CI (95%)	OR	CI (95%)
C18:3 *n*-3	0.792	**0.635–0.988**	0.819	0.642–1.046	0.911	0.766–1.082	0.925	0.772–1.108
C20:5 *n*-3	5.016	0.409–61.557	6.176	0.374–102.027	0.417	0.095–1.835	0.378	0.078–1.823
C22:6 *n*-3	0.830	0.655–1.052	0.833	0.654–1.062	1.033	0.867–1.232	1.033	0.861–1.239
Total *n*-3	0.798	**0.672–0.946**	0.811	**0.675–0.976**	0.959	0.842–1.091	0.968	0.845–1.108
Total *n*-6	1.077	0.986–1.176	1.079	0.984–1.183	1.033	0.975–1.094	1.049	0.987–1.115
Omega-3 index	0.840	0.660–1.069	0.844	0.660–1.079	1.021	0.855–1.220	1.020	0.849–1.226
*n*-6/*n*-3	1.473	**1.021–2.126**	1.421	0.972–2.078	1.099	0.886–1.363	1.117	0.890–1.403
C20:4 *n*-6/C20:5 *n*-3	1.002	0.986–1.020	1.002	0.985–1.020	1.005	0.994–1.016	1.008	0.996–1.019
C18:3 *n*-3/C20:5 *n*-3	0.972	**0.945–1.000**	0.973	0.945–1.003	0.989	0.966–1.012	0.990	0.966–1.014
C18:3 *n*-3/C22:6 *n*-3	0.764	0.455–1.285	0.856	0.482–1.521	0.888	0.599–1.317	0.923	0.613–1.391
C18:2 *n*-6/C18:3 *n*-3	1.276	**1.043–1.561**	1.229	0.995–1.518	0.995	0.980–1.009	0.988	0.970–1.006
Factor 1	1.408	**1.036–1.913**	1.469	**1.056–2.043**	1.208	0.969–1.507	1.276	**1.010–1.612**
Factor 2	1.577	0.878–2.831	1.516	0.812–2.832	1.099	0.876–1.378	1.087	0.860–1.375
Factor 3	0.923	0.671–1.271	0.992	0.697–1.411	0.734	**0.585–0.921**	0.747	**0.589–0.948**

*: model adjusted by body mass index (BMI), physical activity and education level. Omega-3 Index: C20:5 *n*-3 + C22:6 *n*-3. Total *n*-3: C18:3 *n*-3 + 20:5 *n*-3 + 22:6 *n*-3; total *n*-6: C18:2 *n*-6 + C18:3 *n*-6 + C20:2 *n*-6 + C20:3 *n*-6 + C20:4 *n*-6 + C22:2 *n*-6. Odds ratio (OR) per unit change of FA. The bold highlights statistically significant associations.

## Data Availability

Full data can be asked to the corresponding author.

## Supplementary Materials

The following are available online at https://www.mdpi.com/article/10.3390/nu13061919/s1, Table S1: Demographic and clinical characterization of individuals, according to sex. Table S2: Biochemical profile of individuals, according to sex. Table S3: Erythrocyte membranes fatty acids, according to sex. Table S4: Factor loadings of fatty acids in erythrocyte membranes. Table S5: Correlations between erythrocyte membranes PUFA and variables used in cardiovascular risk estimates. Table S6: Multiple linear regressions associating baseline characteristics and erythrocyte membrane fatty acids. Table S7: Multiple logistic regressions associating baseline characteristics and erythrocyte membrane fatty acids. Table S8: Logistic regression models of isolated and pooled erythrocyte membranes fatty acids and ACC/AHA 2013 risk score.

## References

[1] D’Agostino R.B., Vasan R.S., Pencina M.J., Wolf P.A., Cobain M., Massaro J.M., Kannel W.B. (2008). General cardiovascular risk profile for use in primary care: The Framingham heart study. Circulation.

[2] DeGoma E.M., Dunbar R.L., Jacoby D., French B. (2013). Differences in absolute risk of cardiovascular events using risk-refinement tests: A systematic analysis of four cardiovascular risk equations. Atherosclerosis.

[3] Hardoon S.L., Whincup P.H., Lennon L.T., Wannamethee S.G., Capewell S., Morris R.W. (2008). How much of the recent decline in the incidence of myocardial infarction in British men can be explained by changes in cardiovascular risk factors? Evidence from a prospective population-based study. Circulation.

[4] Stamler J., Wentworth D., Neaton J.D. (1986). Is Relationship Between Serum Cholesterol and Risk of Premature Death From Coronary Heart Disease Continuous and Graded?: Findings in 356 222 Primary Screenees of the Multiple Risk Factor Intervention Trial (MRFIT). JAMA.

[5] Unal B., Critchley J.A., Capewell S. (2004). Explaining the decline in coronary heart disease mortality in England and Wales between 1981 and 2000. Circulation.

[6] Kavousi M., Leening M.J.G., Nanchen D., Greenland P., Graham I.M., Steyerberg E.W., Ikram M.A., Stricker B.H., Hofman A., Franco O.H. (2014). Comparison of application of the ACC/AHA guidelines, Adult Treatment Panel III guidelines, and European Society of Cardiology guidelines for cardiovascular disease prevention in a European cohort. JAMA.

[7] DeFilippis A.P., Young R., Carrubba C.J., McEvoy J.W., Budoff M.J., Blumenthal R.S., Kronmal R.A., McClelland R.L., Nasir K., Blaha M.J. (2015). An analysis of calibration and discrimination among multiple cardiovascular risk scores in a modern multiethnic cohort. Ann. Intern. Med..

[8] Cook N.R., Paynter N.P., Eaton C.B., Manson J.E., Martin L.W., Robinson J.G., Rossouw J.E., Wassertheil-Smoller S., Ridker P.M. (2012). Comparison of the framingham and reynolds risk scores for global cardiovascular risk prediction in the multiethnic women’s health initiative. Circulation.

[9] Muntner P., Colantonio L.D., Cushman M., Goff D.C., Howard G., Howard V.J., Kissela B., Levitan E.B., Lloyd-Jones D.M., Safford M.M. (2014). Validation of the atherosclerotic cardiovascular disease Pooled Cohort risk equations. JAMA.

[10] Faludi A.A., Izar M.C., Saraiva J.F., Chacra A.P., Bianco H.T., Afiune Neto A., Bertolami A., Pereira A.C., Lottenberg A.M., Sposito A.C. (2017). Atualização da diretriz brasileira de dislipidemias e prevenção da aterosclerose—2017. Arq. Bras. Cardiol..

[11] Manson J.E., Cook N.R., Lee I.-M., Christen W., Bassuk S.S., Mora S., Gibson H., Albert C.M., Gordon D., Copeland T. (2019). Marine n−3 Fatty Acids and Prevention of Cardiovascular Disease and Cancer. N. Engl. J. Med..

[12] Aung T., Halsey J., Kromhout D., Gerstein H.C., Marchioli R., Tavazzi L., Geleijnse J.M., Rauch B., Ness A., Galan P. (2018). Associations of omega-3 fatty acid supplement use with cardiovascular disease risks meta-analysis of 10 trials involving 77 917 individuals. JAMA Cardiol..

[13] Harris W.S., Von Schacky C. (2004). The Omega-3 Index: A new risk factor for death from coronary heart disease?. Prev. Med..

[14] Chowdhury R., Warnakula S., Kunutsor S., Crowe F., Ward H.A., Johnson L., Franco O.H., Butterworth A., Forouhi N.G., Thompson S.G. (2014). Association of dietary, circulating, and supplement fatty acids with coronary risk. Ann. Intern. Med..

[15] Del Gobbo L.C., Imamura F., Aslibekyan S., Marklund M., Virtanen J.K., Wennberg M., Yakoob M.Y., Chiuve S.E., Dela Cruz L., Frazier-Wood A.C. (2016). ω-3 Polyunsaturated fatty acid biomarkers and coronary heart disease: Pooling project of 19 cohort studies. JAMA Intern. Med..

[16] Harris W.S., Tintle N.L., Etherton M.R., Vasan R.S. (2018). Erythrocyte long-chain omega-3 fatty acid levels are inversely associated with mortality and with incident cardiovascular disease: The Framingham Heart Study. J. Clin. Lipidol..

[17] Willett W.C., Howe G.R., Kushi L.H. (1997). Adjustment for total energy intake in epidemiologic studies. Am. J. Clin. Nutr..

[18] Baecke J.A., Burema J., Frijters J.E. (1982). A short questionnaire for the measurement habitual physical activity in epidemiological studies. Am. J. Clin. Nutr..

[19] Florindo A.A., Latorre M., do R.D., de O. (2003). Validation and reliability of the Baecke questionnaire for the evaluation of habitual physical activity in adult men. Rev. Bras. Med. Esporte.

[20] Garcia L., Osti R., Ribeiro E., Florindo A. (2013). Validação de dois questionários para a avaliação da atividade física em adultos. Rev. Bras. Ativ. Física Saúde.

[21] Masood A., Stark K.D., Salem N. (2005). A simplified and efficient method for the analysis of fatty acid methyl esters suitable for large clinical studies. J. Lipid Res..

[22] Mosca L., Benjamin E.J., Berra K., Bezanson J.L., Dolor R.J., Lloyd-Jones D.M., Newby L.K., Piña I.L., Roger V.L., Shaw L.J. (2011). Effectiveness-based guidelines for the prevention of cardiovascular disease in women—2011 Update: A guideline from the American Heart Association. J. Am. Coll. Cardiol..

[23] Ridker P.M., Buring J.E., Rifai N., Cook N.R. (2007). Development and validation of improved algorithms for the assessment of global cardiovascular risk in women: The Reynolds Risk Score. J. Am. Med. Assoc..

[24] Ridker P.M., Paynter N.P., Rifai N., Gaziano J.M., Cook N.R. (2008). C-reactive protein and parental history improve global cardiovascular risk prediction: The Reynolds risk score for men. Circulation.

[25] Goff D.C., Lloyd-Jones D.M., Bennett G., Coady S., D’Agostino R.B., Gibbons R., Greenland P., Lackland D.T., Levy D., O’Donnell C.J. (2014). 2013 ACC/AHA guideline on the assessment of cardiovascular risk: A report of the American college of cardiology/American heart association task force on practice guidelines. J. Am. Coll. Cardiol..

[26] Catapano A.L., Reiner Ž., De Backer G., Graham I., Taskinen M.R., Wiklund O., Agewall S., Alegria E., Chapman M.J., Durrington P. (2011). ESC/EAS Guidelines for the management of dyslipidaemias. The Task Force for the management of dyslipidaemias of the European Society of Cardiology (ESC) and the European Atherosclerosis Society (EAS). Atherosclerosis.

[27] Landis J.R., Koch G.G. (1977). The Measurement of Observer Agreement for Categorical Data. Biometrics.

[28] Marchioni D.M.L., Latorre M., do R.D., de O., Eluf-Neto J., Wünsch-Filho V., Fisberg R.M. (2005). Identification of dietary patterns using factor analysis in and epidemiological study in São Paulo. São Paulo Med. J..

[29] Winkleby M.A., Jatulis D.E., Frank E., Fortmann S.P. (1992). Socioeconomic status and health: How education, income, and occupation contribute to risk factors for cardiovascular disease. Am. J. Public Health.

[30] Kang H. (2013). The prevention and handling of the missing data. Korean J. Anesthesiol..

[31] Park Y., Lim J., Lee J., Kim S.G. (2009). Erythrocyte fatty acid profiles can predict acute non-fatal myocardial infarction. Br. J. Nutr..

[32] Harris W.S., Kennedy K.F., O’Keefe J.H., Spertus J.A. (2013). Red blood cell fatty acid levels improve GRACE score prediction of 2-yr mortality in patients with myocardial infarction. Int. J. Cardiol..

[33] Kleber M.E., Delgado G.E., Lorkowski S., März W., von Schacky C. (2016). Omega-3 fatty acids and mortality in patients referred for coronary angiography. The Ludwigshafen Risk and Cardiovascular Health Study. Atherosclerosis.

[34] Block R.C., Harris W.S., Reid K.J., Sands S.A., Spertus J.A. (2008). EPA and DHA in blood cell membranes from acute coronary syndrome patients and controls. Atherosclerosis.

[35] Harris W.S., Del Gobbo L., Tintle N.L. (2017). The Omega-3 Index and relative risk for coronary heart disease mortality: Estimation from 10 cohort studies. Atherosclerosis.

[36] Zibaeenezhad M.J., Ghavipisheh M., Attar A., Aslani A. (2017). Comparison of the effect of omega-3 supplements and fresh fish on lipid profile: A randomized, open-labeled trial. Nutr. Diabetes.

[37] Din J.N., Harding S.A., Valerio C.J., Sarma J., Lyall K., Riemersma R.A., Newby D.E., Flapan A.D. (2008). Dietary intervention with oil rich fish reduces platelet-monocyte aggregation in man. Atherosclerosis.

[38] Balk E.M., Lichtenstein A.H., Chung M., Kupelnick B., Chew P., Lau J. (2006). Effects of omega-3 fatty acids on serum markers of cardiovascular disease risk: A systematic review. Atherosclerosis.

[39] Eslick G.D., Howe P.R.C., Smith C., Priest R., Bensoussan A. (2009). Benefits of fish oil supplementation in hyperlipidemia: A systematic review and meta-analysis. Int. J. Cardiol..

[40] Wei M.Y., Jacobson T.A. (2011). Effects of eicosapentaenoic acid versus docosahexaenoic acid on serum lipids: A systematic review and meta-analysis. Curr. Atheroscler. Rep..

[41] DeFilippis A.P., Blaha M.J., Ndumele C.E., Budoff M.J., Lloyd-Jones D.M., McClelland R.L., Lakoski S.G., Cushman M., Wong N.D., Blumenthal R.S. (2011). The association of Framingham and reynolds risk scores with incidence and progression of coronary artery calcification in MESA (multi-ethnic study of atherosclerosis). J. Am. Coll. Cardiol..

[42] Omar Z.A., Montser B.A., Farahat M.A.R. (2019). Effect of high-dose Omega 3 on lipid profile and inflammatory markers in chronic hemodialysis children. Saudi J. Kidney Dis. Transpl..

[43] Miller M., Ballantyne C.M., Bays H.E., Granowitz C., Doyle R.T., Juliano R.A., Philip S. (2019). Effects of Icosapent Ethyl (Eicosapentaenoic Acid Ethyl Ester) on Atherogenic Lipid/Lipoprotein, Apolipoprotein, and Inflammatory Parameters in Patients With Elevated High-Sensitivity C-Reactive Protein (from the ANCHOR Study). Am. J. Cardiol..

[44] Liew S.M., Doust J., Glasziou P. (2011). Cardiovascular risk scores do not account for the effect of treatment: A review. Heart.

