# Supplementary material for: Omega-3 Fatty Acids in Erythrocyte Membranes as Predictors of Lower Cardiovascular Risk in Adults without Previous Cardiovascular Events
Source: Nutrients. 2021 Jun 3;13(6):1919. doi: 10.3390/nu13061919 (PMC8227633; doi:10.3390/nu13061919)
Supplement: Supplementary file 1 [file nutrients-13-01919-s001.zip › nutrients-1212324-supplementary.pdf]

Supplementary materials

Table S1. Demographic and clinical characterization of individuals, according to sex.

Table S2. Biochemical profile of individuals, according to sex.

Table S3. Erythrocyte membranes fatty acids, according to sex.

Table S4. Factor loadings of fatty acids in erythrocyte membranes.

Table S5. Correlations between erythrocyte membranes PUFA and variables used in cardiovascular risk estimates.

Table S6. Multiple linear regressions associating baseline characteristics and erythrocyte membrane fatty acids.

Table S7. Multiple logistic regressions associating baseline characteristics and erythrocyte membrane fatty acids.

Table S8. Logistic regression models of isolated and pooled erythrocyte membranes fatty acids and ACC/AHA 2013 risk score.

Table S1. Demographic and clinical characterization of individuals, according to sex.

Variables	Men	n	Women	n	p-value
Age (years)	49.4 (11.1)	133	54.4 (9.5)	223	**<0.001**
Ethnicity (n)		133		223	0.191
White	87 (24.4)		151 (42.4)		
Non-white	46 (12.9)		72 (32.3)		
Smoking (n)		133		223	**0.003**
Current smoker	35 (26.3)		35 (15.7)		
Non-smoker	98 (73.7)		188 (84.3)		
Alcohol consumption (n)		133		223	**<0.001**
Yes	86 (64.7)		78 (35.0)		
No	47 (35.3)		145 (65.0)		
Education (n)		133		223	0.204
High school or less	72 (54.1)		136 (61.0)		
College	61 (45.9)		87 (39.0)		
Chronic non-communicable diseases (n)		133		223	
Diabetes Mellitus	33 (24.8)		39 (17.5)		0.103
Hypertension	76 (57.1)		127 (57.0)		1.000
Hypothyroidism	3 (2.3)		40 (17.9)		**<0.001**
Dyslipidemia	72 (54.1)		120 (53.8)		1.000
Medication (n)		133		223	
Statins	28 (21.1)		70 (31.4)		**0.037**
Antihypertensives	65 (48.9)		119 (53.4)		0.444
Hypoglicemic	30 (22.6)		44 (19.7)		0.589
Fibrates	3 (2.3)		6 (2.7)		0.549
Family history of diseases (%)		133		223	
Obesity	28 (20.1)		36 (16.1)		0.256
Hypertension	84 (66.2)		148 (66.4)		0.566
Miocardial infarction	38 (28.6)		62 (27.8)		0.903
Stroke	25 (18.8)		43 (19.3)		1.000
Diabetes Mellitus	49 (36.8)		85 (38.1)		0.822
Physical activity (points)	7.2 (1.4)		7.2 (1.4)		0.911
Framingham Risk Score (n. %)		133		223	**0.040**
Low risk	9 (6.8)		34 (15.2)		
Moderate risk	54 (40.6)		73 (32.7)		
High risk	70 (52.6)		116 (52.0)		
Reynolds Risk Score (n. %)		132		219	**<0.001**
Low risk	34 (25.6)		120 (53.8)		
Moderate risk	50 (37.6)		45 (20.2)		
High risk	48 (36.1)		54 (24.2)		
ACC/AHA-2013 Risk Score (n. %)		133		222	**<0.001**
Low risk	27 (20.3)		103 (46.2)		
Moderate risk	16 (12.0)		30 (13.5)		
High risk	90 (67.7)		89 (39.9)		

To determine differences between sex, independent t-test or Mann-Whitney U test were applied to continuous variables. For categorical variables, the X^2-test was applied. For all analyses p-value was set at $p < 0.05$.

Table S2. Biochemical profile of individuals, according to sex.

Variables	Men	n	Women	n	p-value
SBP (mmHg)	134.8 (16.5)	133	132.5 (19.1)	223	0.248
DBP (mmHg)	82.3 (10.2)	133	80.8 (9.7)	223	0.163
BMI (kg/m²)	30.4 (5.4)	133	31.1 (6.0)	223	0.268
Total cholesterol (mg/dL)	198.9 (39.3)	132	208.6 (44.1)	222	**0.039**
LDL-c (mg/dL	131.2 (34.7)	120	140.6 (40.4)	220	**0.032**
HDL-c (mg/dL)	31.0 (27.0-36.0)	132	39.0 (33.0-45.3)	222	**<0.001**
Triglycerides (mg/dL)	151.0 (109.3-221.3)	132	125.0 (91.0-175.8)	222	**<0.001**
Glucose (mg/dL)	100.0 (91.0-115.5)	132	97.0 (91.0-106.0)	222	0.087
Apo A-I (mg/dL)	123.1 (22.2)	132	137.6 (26.2)	223	**<0.001**
Apo B (mg/dL)	102.0 (22.7)	132	105.78 (25.9)	223	0.170
C-reactive protein (mg/L)	2.0 (1.0-5.4)	121	3.3 (1.4-6.4)	226	**0.006**

Continuous variables are shown as mean (standard deviation) or median (interquartile range). To determine differences between sex, independent t-test or Mann-Whitney U test were applied to continuous variables. BMI: body mass index; SBP: systolic blood pressure; DBP: diastolic blood pressure; TG: triglycerides; LDL-c: low density lipoprotein-cholesterol. HDL-c: high density lipoprotein-cholesterol. p-value set at $p < 0.05$.

Table S3. Erythrocyte membranes fatty acids, according to sex.

Variables	Male (n = 125)	Female (n = 210)	p-value
SFA (%)			
C16:0	43.2 (41.3-47.3)	43.8 (41.0-47.7)	0.824
C18:0	24.5 (22.5-26.3)	25.1 (23.2-27.7)	**0.041**
C20:0	0.7 (0.6-0.7)	0.7 (0.6-0.8)	**0.018**
C22:0	1.1 (0.9-1.3)	1.1 (0.9-1.4)	0.179
C24:0	0.3 (0.1-0.7)	0.2 (0.1-0.6)	0.244
MUFA (%)			
C16:1 n-7	0.3 (0.2-0.7)	0.3 (0.2-0.6)	0.982
C18:1 n-9	10.5 (3.2)	9.7 (3.7)	0.076
C20:1 n-9	0.1 (0.1-0.1)	0.1 (0.1-0.1)	0.360
C22:1 n-9	0.1 (0.1-0.2)	0.2 (0.1-0.2)	0.678
C24:1 n-9	1.3 (0.5)	1.3 (0.6)	0.774
PUFA n-6 (%)			
C18:2 n-6	4.9 (1.8)	4.6 (1.8)	0.191
C18:3 n-6	0.2 (0.1-0.2)	0.2 (0.1-0.2)	0.246
C20:2 n-6	0.1 (0.1-0.1)	0.1 (0.1-0.6)	0.422
C20:3 n-6	0.6 (0.3)	0.5 (0.3)	0.305
C20:4 n-6	2.6 (1.5-5.4)	2.4 (1.4-5.1)	0.420
C22:2 n-6	0.4 (0.2-0.5)	0.4 (0.3-0.6)	0.600
Total n-6	9.6 (3.9)	9.2 (3.8)	0.311
PUFA n-3 (%)			
C18:3 n-3	2.0 (1.3-2.5)	1.9 (1.3-2.7)	0.825
C20:5 n-3	0.2 (0.1-0.3)	0.2 (0.1-0.3)	0.815
C22:6 n-3	3.5 (2.8-4.3)	3.2 (2.6-4.1)	0.350
Omega-3 Index	3.7 (3.1-4.6)	3.6 (3.0-4.4)	0.350
Total n-3	5.7 (4.9-6.6)	5.6 (4.6-6.7)	0.373
Fatty acids ratios			
C16:0/C16:1 n-7	130.7 (67.4-231.9)	129.6 (69.1-233.2)	0.881
C18:0/C18:1 n-9	2.3 (1.9-3.1)	2.6 (2.1-3.6)	**0.028**
n-6/n-3	1.6 (1.1-2.5)	1.7 (1.0-2.3)	0.645
C20:4 n-6/C20:5 n-3	14.3 (6.1-27.5)	12.7 (5.1-28.3)	0.630
C18:3 n-3/C20:5 n-3	9.3 (5.6-14.1)	8.5 (5.7-14.0)	0.921
C18:2 n-6/C20:4 n-6	1.7 (1.0-2.9)	1.8 (1.0-2.8)	0.978
C18:2 n-6/C18:3 n-3	2.4 (1.6-4.3)	2.4 (1.4-4.2)	0.486

To determine differences between sex, independent t-test or Mann-Whitney U test were applied to continuous variables. SFA: saturated fatty acids; PUFA: polyunsaturated fatty acids; MUFA: monounsaturated fatty acids-total n-3: C18:3 n-3 + 20:5 n-3+ 22:6. n-6: C18:2 n-6 + C18:3 n-6 + C20:2 n-6 +

C20:3 n-6 + C20:4 n-6 + C22:2 n-6. Data displayed are mean (standard deviation) or median (interquartile range) depending on the distribution. p-value < 0.05 was considered statistical significance.

Table S4. Factor loadings of fatty acids in erythrocyte membranes.

Standardized variables*	Factor 1 (n-6 PUFA)	Factor 2 (neutral)	Factor 3 (n-3 PUFA)
C18:3 n-3	n.s.	n.s.	0.387
C20:5 n-3	n.s.	n.s.	0.732
C22:6 n-3	-0.514	n.s.	-0.434
C18:2 n-6	0.869	n.s.	n.s.
C18:3 n-6	n.s.	0.762	n.s.
C20:2 n-6	-0.412	n.s.	-0.553
C20:3 n-6	0.844	n.s.	-0.209
C20:4 n-6	0.774	-0.236	-0.320
C22:2 n-6	-0.425	n.s.	0.739
C16:1 n-7	n.s.	0.752	n.s.
C18:1 n-9	0.662	0.466	n.s.
C20:1 n-9	n.s.	0.752	n.s.
C22:1 n-9	n.s.	n.s.	n.s.
C24:1 n-9	0.484	n.s.	n.s.
% Explained variance	23.3	15.7	14.6

The cutoff point considered significant for factor loadings was 0.2. n.s.: non-significant loadings. *Factor analysis. KMO = 0.632; Barlett's Test of Sphericity < 0.001.

Table S5. Correlations between erythrocyte membranes PUFA and variables used in cardiovascular risk estimates

**Erythrocyte membranes fatty acids**	**C18:3 n-3**		**C20:5 n-3**		**C22:6 n-3**		**Total n-3**		**Total n-6**		**Omega-3 Index**		**Factor 1**		**Factor 2**		**Factor 3**	
	**r**	**p**	**r**	**p**	**r**	**p**	**r**	**p**	**r**	**p**	**r**	**p**	**r**	**p**	**r**	**p**	**r**	**p**
Age (years)	-0.093	0.089	0.050	0.359	0.002	0.969	-0.064	0.244	0.008	0.878	0.008	0.880	0.028	0.608	-0.009	0.875	-0.057	0.295
Total cholesterol (mg/dL)	-0.155	**0.004**	-0.097	0.077	-0.112	**0.041**	-0.211	**<0.001**	0.178	**0.001**	-0.124	**0.023**	0.170	**0.002**	-0.042	0.443	-0.095	0.083
HDL-c (mg/dL)	0.063	0.251	-0.065	0.233	-0.037	0.496	-0.008	0.879	0.004	0.939	-0.045	0.411	-0.058	0.290	-0.088	0.106	-0.047	0.387
SBP (mmHg)	-0.062	0.261	0.007	0.896	0.090	0.099	0.031	0.571	-0.033	0.547	0.087	0.111	0.012	0.829	0.082	0.133	-0.051	0.354
Glucose (mg/dL)	-0.050	0.357	0.109	**0.046**	-0.004	0.943	-0.021	0.699	-0.135	**0.014**	0.008	0.888	-0.089	0.104	0.143	**0.009**	0.097	0.077
C-reactive protein (mg/L)	-0.068	0.218	0.008	0.885	-0.006	0.910	-0.038	0.496	-0.050	0.369	-0.003	0.960	-0.039	0.477	0.006	0.920	-0.026	0.641

Spearman´s and Pearson´s correlations were tested. p-value < 0.05 was considered statistical significance.

Table S6. Multiple linear regressions associating baseline characteristics and erythrocyte membrane fatty acids.

Covariates*	R^2	β	Confidence interval (95%)
Total n-6 PUFA	0.135		
Constant (β_0)		14.938	**10.79; 19.086**
BMI (kg/m^2)		-0.084	**-0.155; -0.014**
Triglycerides (mg/dL)		0.004	**0.000; 0.009**
Total cholesterol (mg/dL)		0.013	**0.004; 0.023**
Family history of myocardial infarction (yes/no)		-0.807	-1.703; 0.089
Family history of obesity (yes/no)		-1.813	**-2.868; -0.757**
Family history of stroke (yes/no)		-0.954	-1.995; 0.087
Factor 1	0.082		
Constant (β_0)		0.756	-0.247; 1.760
BMI (kg/m^2)		-0.022	**-0.041; -0.003**
Triglycerides (mg/dL)		0.001	**0.000; 0.002**
Total cholesterol (mg/dL)		0.003	**0.001; 0.006**
Family history of myocardial infarction (yes/no)		-0.214	-0.453; 0.025
Family history of obesity (yes/no)		-0.315	**-0.595; -0.036**
Factor 3	0.041		
Constant (β_0)		0.291	-0.589; 1.170
Age (years)		-0.010	**-0.021; 0.000**
Total cholesterol (mg/dL)		-0.002	**-0.005; 0.000**
Family history of obesity (yes/no)		0.399	**0.113; 0.686**

*: Multiple linear regression models resulting from backwards method. The initial model included the covariates age, sex, race, schooling, smoking, systolic blood pressure, BMI, glucose, triglycerides, total cholesterol, HDL-c, C-reactive protein, physical activity, drinking habits, treatments with statins, antihypertensives, fibrates, and hypoglycemic drugs, family history of myocardial infarction, obesity, hypertension, and stroke. Total n-6 PUFA, Factor 1 and Factor 3 were used as dependent variables.

Table S7. Multiple logistic regressions between baseline characteristics and erythrocyte membrane fatty acids.

Covariates*	OR	Confidence interval (95%)
Total n-3 PUFA†		
Constant (β_0)	0.304	
Sex (male/female)	1.665	0.921; 3.009
Schooling (complete/incomplete)	0.693	0.421; 1.143
Smoking (yes/no)	1.457	0.761; 2.789
SBP (mmHg)	1.013	0.999; 1.027
BMI (kg/m^2)	1.053	**1.006; 1.102**
Triglycerides (mg/dL)	0.996	**0.993; 0.999**
Total cholesterol (mg/dL)	0.995	0.989; 1.002
HDL-c (mg/dL)	1.016	0.988; 1.046
Physical activity (points)	0.909	0.765; 1.080
Alcohol consumption (yes/no)	0.712	0.420; 1.208
Antihypertensive treatment (yes/no)	0.752	0.448; 1.261
Hypoglycemic treatment (yes/no)	0.575	0.305; 1.082
Family history of obesity (yes/no)	0.689	0.372; 1.275
Factor 2††		
Constant (β_0)	7.676	
Smoking (yes/no)	1.183	0.629; 2.227
SBP (mmHg)	0.997	0.984; 1.010
Total cholesterol (mg/dL)	0.998	0.992; 1.004
HDL-c (mg/dL)	0.982	0.957; 1.007
C-reactive protein (mg/L)	1.008	0.973; 1.045
Physical activity (points)	0.947	0.798; 1.124
Alcohol consumption (yes/no)	0.826	0.503; 1.355
Statin treatment (yes/no)	0.914	0.536; 1.560
Fibrate treatment (yes/no)	1.867	0.436; 7.986
Hypoglycemic treatment (yes/no)	0.852	0.462; 1.570
Family history of myocardial infarction (yes/no)	0.903	0.537; 1.516
Family history of obesity (yes/no)	0.323	**0.166; 0.629**
Family history of hypertension (yes/no)	1.288	0.779; 2.131

*: Multiple logistic regression models resulting from backwards method. The initial model included the covariates age, sex, race, schooling, smoking, systolic blood pressure, BMI, glucose, triglycerides, total cholesterol, HDL-c, C-reactive protein, physical activity, drinking habits, treatments with statins, antihypertensives, fibrates, and hypoglycemic drugs, family history of myocardial infarction, obesity, hypertension, and stroke. Total n-3 PUFA and Factor 2 were used as dependent variables. †: Correct classification = 65.0%; Specificity = 65.2%; Sensibility = 64.8%; Hosmer-Lemeshow p-value = 0.150; Nagelkerke R^2 = 0.121. ††: Correct classification = 63.4%; Specificity = 52.3%; Sensibility = 74.4%; Hosmer-Lemeshow p-value = 0.062; Nagelkerke R^2 = 0.085.

Table S8. Logistic regression models of isolated and pooled erythrocyte membranes fatty acids and ACC/AHA 2013 risk score.

Fatty acids	ACC/AHA 2013 Risk Score			
	Unadjusted model		Adjusted model*	
	OR	Confidence interval (95%)	OR	Confidence interval (95%)
C18:3 n-3	0.926	0.779-1.102	0.951	0.791-1.142
C20:5 n-3	0.726	0.163-3.243	0.678	0.137-3.343
C22:6 n-3	0.965	0.809-1.152	0.976	0.813-1.172
Total n-3	0.936	0.821-1.068	0.956	0.833-1.097
Total n-6	1.005	0.948-1.065	1.009	0.949-1.072
Omega-3 index	0.960	0.802-1.149	0.971	0.807-1.168
n-6/n-3	1.006	0.811-1.247	0.983	0.785-1.231
C20:4 n-6/C20:5 n-3	0.997	0.986-1.008	0.998	0.987-1.010
C18:3 n-3/C20:5 n-3	0.980	0.957-1.004	0.982	0.958-1.006
C18:3 n-3/C22:6 n-3	1.023	0.682-1.536	1.082	0.706-1.659
C18:2 n-6/C18:3 n-3	1.004	0.988-1.021	0.999	0.983-1.015
Factor 1	1.142	0.915-1.424	1.168	0.925-1.475
Factor 2	1.115	0.875-1.419	1.095	0.851-1.408
Factor 3	0.866	0.693-1.083	0.897	0.708-1.136

*: model adjusted by body mass index (BMI). physical activity and education level. Omega-3 Index: C20:5 n-3 + C22:6 n-3. Total n-3: C18:3 n-3 + 20:5 n-3+ 22:6 n-3; total n-6: C18:2 n-6 + C18:3 n-6 + C20:2 n-6 + C20:3 n-6 + C20:4 n-6 + C22:2 n-6.Odds ratio (OR) per unit change of FA.
